# Before the Lords' Committee

**Published:** 1890-06-14

**Authors:** 


### Before the Lords' Committee.

(By Our Special Commissioner.)

### III.—Mr. Timothy Holmes, Mr. Nelson Hardy, and Mr. Hugh Woods.

### Out-Patients' Departments.

The evidence given by Mr. Timothy Holmes, Surgeon to St. George's Hospital, dealt chiefly with Provident Dispensaries in relation to hospital out-patient departments. There are one or two special points which Mr. Holmes incidentally mentioned to which we may profitably direct attention. He stated, for instance, that there was no out-patient department one hundred years ago in any hospital in England. Certainly there was not one at St. George's or St. Bartholomew's. Now, as a matter of fact, patients have been seen at St. Bartholomew's Hospital for more than two centuries, and at St. George's, Mr. Holmes' own hospital, a reference to the report will show that both in and out patients have been treated there ever since 1733, although no particular analysis of in or out patients was kept until the year 1803, when the out-patients averaged some 600 per annum. They now amount to 26,500. Besides there has been an out-patient department attached to the London Hospital from the date of its foundation in 1740. Out-patients have been seen at the Leeds Infirmary from the date of its establishment in 1768, and at the Hereford General Infirmary the same practice seems to have prevailed since the date of its institution in 1776. We have only quoted the above as representative cases of various classes of hospitals in London and the provinces, but very many similar institutions might be mentioned in correction of the error into which Mr. Holmes fell on this point.

### Paris Hospitals.

Mr. Holmes is also in error in stating that the Paris hospitals are supported by an administration called the "Administration de l'Assistance Publique," which is a branch of the Paris Municipality; the property of the hospitals meeting about a quarter of their expenses, and three-fourths being paid by the "Administration de l'Assistance Publique." This is not so, however. In the first place the "Assistance Publique" was organized by the Law of January 10th, 1849. It was placed under the special supervision of the Minister of the Interior, being in the jurisdiction of the Prefect of the Seine, and was administered by a responsible Director, aided by a superior Council of Inspection, whose composition and duties were determined by the Decree dated April 24th, 1849. It was charged with the double mission of, (a) providing treatment for the sick and wounded, and relief of the aged, infirm, and all who are incapable of self-support; and (b) affording medical advice in their own homes to those who have to remain with their families. The Director is invested with authority to regulate its interior and exterior organization. He prepares the budget and accounts for the expenditure, and makes periodical reports to the Municipal Council of Paris of the results of his administration. The Council of Inspection supervises the budget and accounts, as well as the statement of expenditure presented by the Director; is charged with the duty of managing all the property of the "Assistance Publique"; and has to decide upon all plans, contracts, the acceptance or refusal of gifts and legacies, the investment of funds, and all other business transactions. The Council is responsible for the conduct of the directors, surgeons, and chemists, and the staff generally, subject to the Minister of the Interior, who has power to cancel any appointment on a vote of the Council presented through the Prefect. The revenues of the "Assistance Publique" are derived (1) from various properties and investments, which produce about six million francs annually; (2) from taxes, including those on theatre, ball, and concert tickets, amounting altogether to a sum of five million francs; (3) from patients' payments, which yield about four million francs; (4) from sales, legacies, and other miscellaneous receipts. Any deficiency between the revenue derived from these sources and the actual expenditure is made good each year by a subsidy from the Municipality of Paris. Some idea of the working of this system may be gathered from the statement that the expenditure in 1888 amounted to 40,714,000 francs, which was met as follows:—By the revenues of the "Assistance Publique," as above enumerated, 18,118,400 francs; by subsidy supplied by the Paris Municipality, 22,595,600 francs. It will thus be seen that the Municipality contributed rather more than one-half of the necessary expenditure.

### A General Practitioner's Evidence.

Mr. Nelson Hardy, a general practitioner, made a somewhat fierce attack upon the hospital system, but the force of his evidence was weakened by an absence of precision, which lessened the effect of much of what he said. He extolled the excellence of the free dispensary system as opposed to the out-patient system of St. Bartholomew's Hospital, but admitted that he spoke from general knowledge mainly, and that at the dispensary not more than four minutes were devoted to each patient. His attack was mainly levelled against the three endowed hospitals—Guy's, St. Bartholomew's, and St. Thomas's; and he at first stated that he believed sixty patients an hour were seen, as a rule, at St. Bartholomew's Hospital, though he subsequently admitted that his evidence was derived partly from personal inspection, partly from hearsay, and partly from reports in the newspapers. We mention this in justice to Mr. Hardy, as his evidence is too voluminous to give in full, and we have only space for one of several instances where his want of practical knowledge led him seriously astray. He stated that sometimes on a Monday a thousand out-patients would be seen at St. Bartholomew's by a staff of twelve medical men. So far from this being the case, it appears the total number of the staff engaged every morning in seeing out-patients at St. Bartholomew's Hospital is as follows: In the surgery twenty members of the senior and junior staff with forty-four students, and in the out-patient department two members of the staff and sixteen students. That is to say, there are in reality eighty-two medical men, including students, engaged in the out-patient department, instead of twelve, as stated by Mr. Hardy, and these eighty-two do not include the physicians and surgeons in charge of the special departments. It is reasonable to ask that subsequent witnesses shall take more care to ascertain the facts before tendering evidence for the information and guidance of the Lords' Committee. No one sympathizes more than we do with the just grievances which medical practitioners have against the out-patient system at present prevailing at the hospitals. They will, however, destroy their case altogether, if they do not take care to send representatives to give evidence who can substantiate every point which they advance in support of the objections which they may justly urge against the present system. Much of Mr. Hardy's evidence was useful as giving a *résumé* of what is somewhat ancient history now—the attempts to secure a reform in the out-patient department. For instance, Mr. Hardy made a point of the old bad ways of certain second-class hospitals, who used to return the attendances, and not patients treated, as evidence of work done. This system was exposed long ago, and is now recognised as disgraceful by all respectable hospital authorities. Besides, the amended form in which returns are now made to the Hospital Sunday Fund has stopped the practice effectually, so far as the institutions which receive grants from it are concerned, at any rate. We should also like to ask Mr. Hardy if he has ever seen the form of prescription-papers and cards which are issued from poor-law dispensaries to patients attending them. If he has not, we would suggest he should procure one, in order that he may realize how injurious and impossible it would be to compel every poor person who cannot pay a doctor to go without medical aid unless they sought it at the poor-law dispensary. There is something, however, to be said for Mr. Hardy's suggested rule, that no out-patient should be treated at the hospital unless he brought with him a note from some medical practitioner.

An Amusing Picture.

Mr. Hardy deserves credit for his picture of the hospital out-patient with a pint bottle of physic prescribed for by the first physicians in London, but, alas! "containing mostly Epsom salts." If this be true, the mechanism of our outpatient departments must resemble a steam hammer which is so adjusted that it can as readily crack a nut as strike a thousand ton blow.

Mr. Hugh Woods, M.D., B.S.

The Committee seem to have been surprised after hearing a portion of the evidence tendered by Mr. Woods, that he should have presented himself as a witness, and well they might be. He stated that he was an Irish medical practitioner, educated in Dublin, who had been in this country for three years, the first of which he spent as an assistant to a medical practitioner in Wales. During the last two years he had been in practice at Highgate, where he stated that he takes fees as low as a shilling. He admitted that he had had no personal experience of provident hospitals or provident dispensaries, and that he had never lived in any of the London hospitals. Pressed by the Chairman as to whether he had had any practical experience, he urged that he had had the benefit of a great deal of practical experience from many others. The Chairman, however, justly insisted that Mr. Hugh Woods had had no practical experience of the general hospitals of London, and he, therefore, asked him to confine his evidence to the effect of free hospitals upon general practitioners. Though brought to his bearings at the outset Mr. Hugh Woods' evidence affords much amusing reading to the initiated, who were, however, not unprepared for such an anti-climax to the various attacks and reflections on the hospitals which have appeared in the newspapers over the name of Mr. Hugh Woods. The truth is, as we said above, the general practitioners must do better than this or the Committee will be forced to conclude either that they have no case, or that their onslaught upon the hospitals is, at the best, "very small potatoes."

